# Targeting Microbiota: What Do We Know about It at Present?

**DOI:** 10.3390/medicina55080459

**Published:** 2019-08-10

**Authors:** Aleksejs Derovs, Sniedze Laivacuma, Angelika Krumina

**Affiliations:** 1 Riga Stradins University, Department of Internal Medicine, LV-1007 Riga, Latvia; 2 Riga East Clinical University Hospital, LV-1038 Riga, Latvia; 3 Riga Stradins University, Department of Infectology and Dermatology, LV-1007 Riga, Latvia

**Keywords:** gut, microbiome, liver disease, CNS, neurodegenerative disease, probiotics

## Abstract

The human microbiota is a variety of different microorganisms. The composition of microbiota varies from host to host, and it changes during the lifetime. It is known that microbiome may be changed because of a diet, bacteriophages and different processes for example, such as inflammation. Like all other areas of medicine, there is a continuous growth in the area of microbiology. Different microbes can reside in all sites of a human body, even in locations that were previously considered as sterile; for example, liver, pancreas, brain and adipose tissue. Presently one of the etiological factors for liver disease is considered to be pro-inflammatory changes in a host’s organism. There are lot of supporting data about intestinal dysbiosis and increased intestinal permeability and its effect on development of liver disease pointing to the gut–liver axis. The gut–liver axis affects pathogenesis of many liver diseases, such as chronic hepatitis B, chronic hepatitis C, alcoholic liver disease, non-alcoholic liver disease, non-alcoholic steatohepatitis, liver cirrhosis and hepatocellular carcinoma. Gut microbiota has been implicated in the regulation of brain health, emphasizing the gut–brain axis. Also, experiments with mice showed that microorganisms have significant effects on the blood–brain barrier integrity. Microbiota can modulate a variety of mechanisms through the gut–liver axis and gut–brain axis. Normal intestinal flora impacts the health of a host in many positive ways, but there is now significant evidence that intestinal microbiota, especially altered, have the ability to impact the pathologies of many diseases through different inflammatory mechanisms. At this point, many of the pathophysiological reactions in case of microbial disbyosis are still unclear.

## 1. Introduction

The human microbiota is a variety of different microorganisms that live within and on most human bodies. Microbiota is diverse between different hosts and it changes during a lifetime. It is known that the microbiome may be changed because of diet, bacteriophages and different processes, for example, inflammation [1,2,3].

Like all other areas of medicine, there is a continuous growth in the area of microbiology. In the last decade, there have been thrilling achievements in the fields of microbiota and its diversity as a result of different positive and negative factors. Microbiota can act on human and environmental targets through different substances. The most intriguing ones among them are antibiotics. There is a large amount of data about soil microorganisms producing antibiotics, but very little information about production of these substances in human microbiota [1,4].

There is an abundant number of bacteria in most humans. Moreover, there are estimates that the number of bacteria may be equal to cells of a person [5]. Most of these microbes reside in the gastrointestinal system where more than 1000 species are represented [6]. Bacteria present in gut also help humans, for example, by fermenting non-digestible compounds, such as starch. Microorganisms living in the gut also provide different substances, for example, several short-chain fatty acids like formate, acetate, propionate and butyrate. Many flavanol compounds like kaempferol, quercetin, myricetin are metabolized by *Clostridium* species microorganisms and then these polyphenol compounds can be transformed and moved to human cells to exhibit many beneficial effects. Intestinal microbiota also provides some of micronutrients such as riboflavin, biotin, and vitamins B, A, D and K [7,8].

Pathological changes in the gut microbiota (microbial dysbiosis) and sometimes abundance of certain bacterial strains, have been known to have an effect in different diseases, such as diabetes, cardiovascular disease, liver disease, inflammatory bowel disease, irritable bowel syndrome, multiple sclerosis, autism, Alzheimers disease, Parkinson’s disease and amyotrophic lateral sclerosis. All of these diseases have unique characteristics but one of the common defining features is chronic inflammation. Previous studies argued that it is a result of microbial dysbiosis [8,9].

## 2. Microorganisms in Different Body Sites

Different microbes can reside in all sites of a human body, even in locations that have previously been thought to be sterile, for example, liver, pancreas, brain and adipose tissue. Microorganisms in different body sites are capable of producing metabolites to protect the host.

### 2.1. Microbes in Oral Cavity and Upper Airways

In the oral cavity, microorganisms can live in a number of sites including tongue, gums, teeth and oropharyngeal areas. The oral cavity can be considered to be a habitat for more than 700 microorganism species. All of these microbes interact among themselves and with host cells throughout different signaling systems [1,10,11].

The human microbiome has unique biosynthetic clusters that do not have anything similar elsewhere in nature. For example, there is network consisting of *Candida albicans* and *Streptococcus mutans*, *Pseudomonas aeruginosa* and prokaryotes where *Streptococcus mutans* secretes non-ribosomal substances like mutanobactins and mutanamide, that disturbs *Candida albicans* hypha that are required for an invasion thus reducing possibility of thrush development [1,12]. These mutanobactines have an effect on different cytokines especially on IL-6 and IL-12 which have pro-inflammatory activity. However, the mutanobactine molecular targets are unknown. Also previously mentioned *Streptococcus mutans* produces lantibiotics, mutacin 1140 and mutacin -B-NY266 that have a broad spectrum of activity against Gram-negative microbes [1,13]. As a further example, we discuss a relationship between *Streptococcus salivarius* and *Streptococcus pyogenes. Streptococcus salivarius* is the predominant commensal in oral cavity, and it produces different lantibiotics (like salivaricins B and D) that protects from invasion of bacteria like *Streptococcus pyogenes*, *Micrococcus luteus* and *Corynebacteium* species [1,14,15,16].

Experiments in mice models showed that *Staphylococcus saprophyticus* may sense a pathogen and initiate a response with unidentified molecules which are then sent to *Streptococcus infantis*. Subsequently, *Streptococcus infantis* signals to *Streptococcus sanguinis* which produces hydrogen peroxide to kill pathogenic *Escherichia coli* species [17].

### 2.2. Microbes in Gastrointestinal Tract

Microbiome in gastrointestinal tract is very rich in different microorganism species, and it has been referred as “the forgotten organ” or “virtual metabolic organ”. The predominant species are *Ruminococci*, *Bifidobacteria*, *Proteobacteria*, *Lactobacilli*, *Streptococci* [18]. Gut commensals secrete chemical signals that modulate the mucosal immunity and inflammatory response as a strategy to fight different pathogens.

For example, *Bacteroides fragilis*, a prominent resident of the gastrointestinal system, produces immunomodulatory and pro-inflammatory polysaccharides like polysaccharide A. This polysaccharide may induce a cascade of processes that results in production of IL-10 and promotes colonization of *Bacteriodes fragilis*, but simultaneously restricts colonization of other possible competitors such as *Helicobacter hepatis* that is important in development of colitis. There are reports from experiments with animals that even oral administration of this polysaccharide may reduce the risk of developing colitis [1,19,20].

Another example of species, which employs the abovementioned strategy, is *Lactobacillus plantarum* that produces pyro-peptides. These compounds decrease levels of pro-inflammatory cytokine interferon (INF)-gamma and thus exhibit immunomodulatory effects [21].

Gut microbiota plays the primary role in host immune responses, and it enhances the production of different antimicrobial compounds that help to fight pathogens. In experiments with mice, removing gut microorganisms results in an increased susceptibility to infectious agents such as influenza virus or lymphocytic choriomengitis virus [1,22]. Other gastrointestinal tract residents like *Bifidobacterium longum* and *Bacteroides thetaiotaomicron* are able to metabolize arachidonic acid and produce prostaglandins and leukotrienes which affect immune response and the ability to fight infectious diseases [23].

Gut microorganisms are known to produce bacteriocins that can kill closely related bacterial species and confer an ecological advantage on the producer. For example, *Lactobacillus gasseri* produce gassericin B which has antibacterial activity against *Listeria monocytogenes*, *Staphylococcus aureus* and *Bacillus cereus*. *Lactobacillus reuteri* produces reuterin that has the ability to protect the host against *Escherichia coli* [1]*. Lactobacillus plantrum* produces lantipeptides called plantaricins that have activity against Gram-positive bacteria such as *Streptococcus thermophilus* and *Enterococcus fecalis* [24]. *Pediococcus* produces pediocins that are effective against *Listeria* species [25]. Emphasizing the role of intestinal microbiota, intestinal *Escherichia coli* produces wide spectrum of microcins lie MccS, MccC7, MccJ25, MccB17, and MccH47. For example, MccC7 has inhibitory activity against common pathogenic microorganisms like *Klebsiella*, *Salmonella*, *Shigella*, *Yesinia* and *Proteus*. *Bacillus thurigiensis* produces thuricin CD that has a potent effect against *Clostridium difficile* and *Listeria monocytogenes*. *Bacillus subtilis* produces amicoumacin A that has antibiotic and anti-inflammatory effect. It is useful in a fight against *Helicobacter pylori* [26,27].

The production of different ferments is not the only a possibility of demonstrating antimicrobial activity. Lactic acid producing microorganisms (*Lactobacilli* and *Bifidobacteria*) are able to protect the host from pathogens by hindering of pathogenic microorganisms’ adhesion on the gut mucosa. The adherence factors on the probiotic cell surface are also known to prevent the attachment of some pathogens. For example, some surface layer proteins in different species of *Lactobacillus* prevent from *Salmonella enterica* and *Echerichia coli*, as a result of a competition between pathogen and probiotic for the same receptor. Additionally, the inhibition of pathogenic toxins is yet another way of protecting a human organism; it is a reason why some probiotics are effective in the cases of diarrhea [28]. Moreover, the probiotics help to save the balance of microorganisms in the gastrointestinal tract, being important because of limited nutrients in gut. If probiotic microorganisms occur in a normal count, it is harder for pathogens to compete with them for sustenance.

### 2.3. Microbes in Genitourinary Tract

The vaginal microbial ecosystem is relatively unstable and can rapidly change even during one day. The flora mainly consists of lactic acid-producing microorganisms. It is widely known that a shift in the vaginal flora in the case of a reduction on *Lactobacilli* and increase in other organisms may directly lead to bacterial vaginosis and even pelvic inflammatory disease in the long term [29].

*Lactobacilli* have a number of strategies against different pathogens. Several members of the *Lactobacillus* family produce bio-surfactants composed of lipids, proteins and carbohydrates. These bio-surfactants repel pathogens, destroy their biofilms and stop colonization. For instance, *Lactobacillus reuteri* and *Lactobacillus iners* can destroy biofilm of *Gardnerella vaginalis* [30,31].

### 2.4. Microbes on Skin

The human skin can have highly non-uniform microbiota. There are microbes in deeper layers of the skin and even in the adipose tissue which were previously considered sterile. Numerous evidences show that the skin microflora is directly linked to the immune system [32]. The most dominant microorganism families on skin are *Staphylococcus*, *Propionibacterium*, *Brevibacterium*, *Corynebacterium*, *Micrococcus*, *Streptococcus* [1,33].

*Staphylococcus epidermidis* and *Priopionibacterium acne* compete with each other to inhabit similar skin niches and they have potent antimicrobial activity against each other. There are data that *Staphylococcus aureus* may even cross boundaries between different kingdoms and fight skin parasite *Leischmania major* [34]. *Staphylococcus epidermidis* also is able to degrade biofilms of other pathogens like *Staphylococcus aureus* by producing lytic enzymes such as serine proteases [1].

### 2.5. Identification of Gut Microbiota

There are two major options to determine gut microorganisms: the stool culture method for a bacteriological analysis and a genetic analysis. However, none of these methods is ideal. Stool culturing is widely used, especially for a diagnostic of known gut pathogens, which grow on a specific substrate, but it is not enough for an evaluation of the full pattern of gut microbiota, and particularly identifying its diversity. For this purpose, genetic testing is needed to ensure accuracy in qualitative and quantitative investigations. Genetic testing allows us to identify different bacteria groups and their proportion. Different techniques are employed for this purpose, such as polymerase chain reaction (PCR), fluorescence in situ hybridization (FISH), microarray DNA technology (also based on hybridization). Such technologies allow us to generate a taxonomic tree of multiple different bacterial species. However, these methods are expensive and are not widely available [35].

## 3. Diseases and Dysbiotic Features

There are also many possible associations with diseases and features of dysbiosis that are summarized in table below (Table 1) [36,37,38,39,40,41,42,43,44,45,46,47,48,49,50,51,52,53,54,55,56,57,58,59,60,61,62,63,64,65,66,67,68,69,70,71,72,73,74,75,76,77,78,79,80,81].

### 3.1. Effect of Microbiome on Gut–Liver Axis

Presently, one of the etiological factors for liver diseases is considered to be pro-inflammatory changes in the host’s organism. Intestinal dysbiosis and increased intestinal permeability have an effect on development of liver disease, supporting the existence of gut–liver axis [36].

Liver and the intestinal tract interact with each other by links through the biliary tract, portal vein and systemic circulation. This interaction is ensured by production of bile acids and different bioactive mediators into both, the biliary system and the main circulation. In the intestine, microorganisms transform acids (like bile and amino acids) and other substances from diet and outer environment. Afterwards, they are translocated to the liver by the portal vein and thus influence processes in the liver [37].

Bile acids and intestinal microorganisms interact very closely and continuously modulate each other. It is argued that bile acids have direct control over intestinal microbiota [38]. For instance, intestinal dysbiosis shifts the alignment between the primary and secondary bile acids and their enterohepatic circulations. On the other hand, bile acids are involved in inhibiting intestinal bacterial overgrowth processes [39,40,41].

The gut–liver axis affects pathogenesis of many liver diseases, such as chronic hepatitis B, chronic hepatitis C, alcoholic liver disease, non-alcoholic liver disease, non-alcoholic steatohepatitis, liver cirrhosis and hepatocellular carcinoma [42].

### 3.2. Non-Alcoholic Liver Disease (NAFLD)

NAFLD refers to two different conditions: non-alcoholic fatty liver which is mostly just an accumulation of fat in the liver, and non-alcoholic steatohepatitis (NASH) which besides the fat accumulation presents an inflammation, and thus, is a progressive liver disease that may lead to cirrhosis and liver malignancies.

There are several studies that refer to a possible connection between NAFLD and intestinal microbiota [43]. For instance, patients with NALFD have a higher prevalence of small intestinal bacterial overgrowth and microbial dysbiosis. In these patients, there is an increased amount of *Bacteroides* and *Ruminococcus*, but a decreased number of *Prevotella.* There are higher amounts of *Escherichia coli* and *Bacteroides vulgatus* in patients with fibrosis in stages 3 and 4. Several studies described enrichment of *Escherichia* in obese children with NASH in comparison with obese children without NASH [44].

Several studies have pointed out a possibility to use some metabolites for early diagnostics of NASH. For example, there is a study that suggests that patients with NASH have an increased microbial production of ethanol [45]. In addition, NAFLD patients have increased levels of systemic trimethylamine N-oxide (TMAO) and liver bile acid synthesis and decreased production of phosphatidylcholine [36,41]. The intestinal microflora and its products may be used as markers for NAFLD. The possibility to affect these microbes has a potential for treating or at least managing NAFLD [36].

### 3.3. Alcoholic Liver Disease

The manifestation of liver damage in alcohol abusers is affected by different factors, including genetics, immune system, environmental factors and intestinal microbiome [46]. Only 15–20% of patients with alcohol overuse develop alcoholic liver disease, and these changes are largely connected to microbiota [47].

Like NAFLD, small intestinal bacterial overgrowth is occasionally observed in alcohol associated liver disease both, in human and experimental mouse models. In patients with alcohol abuse, an increase of *Enterobacteriaceae* family and reduction in *Lactobacillus* and *Bacteroides* is observed [48,49]. Alcohol abuse also reduces taxa that produce short-chain fatty acids such as *Lachnospiracea* and *Ruminococcaceae* [50]. Furthermore, *Fusobacteria* numbers were higher for patients who drink alcoholic beverages than for teetotalers. Nonetheless, the numbers were lower than for those with a moderate alcohol intake [46].

Previous studies showed that stool transfer from drinking human to germ-free mice leads to bacterial translocation. However, hepatic inflammation and cirrhosis does not develop unless mice are fed alcohol [51]. In comparison with NAFLD, where the process is believed to be irreversible, alcohol induced dysbiosis is partially reversible [52]. It has also been observed that people who use alcohol have a reduced fungal diversity, and they may have *Candida* overgrowth [53].

In case of an alcohol-induced intestinal barrier impairment and bacterial translocation, TLRs and other elements recognize pathogens and activate Kupffer cells. This process initiates cascades releasing TNF-alfa and interleukins like IL-1, IL-10, IL-12 and TGF-beta [36,54].

In studies with rodents, alcohol-related gut permeability changes are connected to a microbiota alteration by reduction of intestinal hypoxia-induced factor 1 alfa [55]. Several studies point to intestinal permeability in alcohol abusers, which was linked to higher prevalence of depression, anxiety and desire for alcohol even after it was withdrawn [46]. Furthermore, problems with achieving disease remission in cases of alcohol hepatitis are observed, because alcohol abstinence has success rate of 20–40% of early remission [56].

As there is an increasing amount of data about the role of microorganisms in the pathogenesis of alcohol-related liver disease, fecal microbiota transplantation is explored as one of the potential treatment methods. In one study it even led to the conclusion that it may significantly improve 1-year outcome in steroid-resistant alcoholic hepatitis [57]. The study with administration of probiotics (*Bifidobacterium bifidum* and *Lactobacillus plantarum* 8PA3) for 5 days in patients with mild alcohol-induced hepatitis, showed a notable improvement of liver enzymes (ALT, AST, GGT) [52].

### 3.4. Cirrhosis

Cirrhosis is end-stage liver disease with a significant reduction in active hepatocytes, thick fibrous scarring and regenerating nodules.

Intestinal microbiota has a significant impact on complications of liver cirrhosis. For example, a treatment of portal systemic encephalopathy includes treatment with non-systemic antibiotics to reduce intestinal microbial overgrowth [58].

There are studies that implicate that patients with liver cirrhosis have changed microbiomes. They have an overrepresentation of microorganism genera including *Veilonella*, *Megasphera*, *Dialister*, *Atopobium* and *Prevotella* in *duodenum*. Also, a presence of *Neisseria* and *Gemella* is discriminative between hepatitis B and PBC related cirrhosis [59].

In a specific study, patients with alcoholic cirrhosis who quit drinking still had worse gut dysbiosis and endotoxemia than those who do not drink and do not have alcohol-related cirrhosis [60]. In patients with alcoholic cirrhosis, increased numbers of *Enterobacteriaceae* and decreased numbers of *Lachnospiraceae* and *Ruminococcceae* species are observed [61]. Patients with alcohol-related cirrhosis have higher amounts of the oral flora in feces. Such an observation was explained by higher rates of changes in oral cavity, i.e., periodontitis, transformation in salivary microbiota, a use of proton pump inhibitors and lower gastric acid levels [62].

There are data that connect the use of non-absorbable antibiotics with a decrease of liver fibrosis. Furthermore, there are suggestions that the *Lactobacillaceae* strain containing probiotical products could potentially improve disease outcome in patients with alcohol-related cirrhosis [63].

### 3.5. Hepatocellular Carcinoma (HCC)

The intestinal microbiota is dramatically different in hosts who have HCC. For example, it is shown in mouse models that HCC is accompanied with enriched *Clostridium* bacteria populations. Furthermore, it is found that they have an *Escherichia coli* overgrowth [64]. Mice models also implicate a migration of *Helicobacter* species to HCC tumor tissue [65].

The available data show a connection between an application of broad-spectrum antibiotics and attenuating of liver inflammation leading to HCC development in mice [36].

### 3.6. Effect of Microbiome on CNS

Gut microbiota is involved in different processes involved in brain health and functioning, emphasizing the gut–brain axis. For example, decarboxylases secreted by *Clostridium sporogenes* are important in the conversion of tryptophan to tryptamine which is a neuromodulator and neurotransmitter that evokes release of serotonin and dopamine [66]. Also, the *Lactobacillus* and *Bifidobacteria* species can convert glutamate to the neurotransmitter-gamma-aminobutyric acid [67].

Experiments with mice showed that microorganisms have a significant effect on blood–brain barrier integrity by regulating the junction proteins occludine and claudin 5 because the production of these proteins in germ-free mice was reduced by up to 75% [68].

Microbiota is able to interact with CNS using different mechanisms, like vagal nerve or by transmitting signaling molecules through the circulatory system [69].

### 3.7. Neurodegenerative Diseases

Amyotrophic lateral sclerosis, Parkinson’s disease and Alzheimer’s disease and many other diseases are characterized by a progressive neuronal loss, cognitive decline and impaired motor function [69].

Animal studies provide information about the fact that a healthy and diverse gut microflora may impact brain functioning and health. It has been shown that microorganisms of the *Lactobacillus* species have anti-inflammatory effects in rat models [70].

### 3.8. Amyotrophic Lateral Sclerosis (ALS)

ALS is characterized by progressing neurodegeneration and muscular weakness.

In ALS patients, a reduced membrane integrity of the blood–spinal cord barrier and blood–brain barrier is observed. A leakage of an enhanced blood–brain barrier is accompanied with elevated immune cell infiltration and intensified CNS inflammation. For example, such a leakage causes an increase in concentration of mast cells and macrophages in the spinal cord. Furthermore, upregulated COX-2 levels are observed [69,71].

Models show that in patients with ALS, there are decreased amounts of fecal *Escherichia coli*, *Firmicutes peptostreptococcus* and *Butyrivibrio fibrisolvens* [72].

The accumulated data indicate that the ALS pathology is driven by changes of the gut microbiota and even its diversity, which, in turn, can initiate a systemic inflammation, as well as a weakening of intestinal and CNS barriers providing it with imperfections [69].

### 3.9. Parkinson’s Disease

It is known that Parkinson’s disease has several cellular and molecular characteristics, but it was suggested that the intestinal microbiota plays a significant role in its development as well. Patients with Parkinson’s disease frequently have a microbial overgrowth, with an excessive increase in coliform bacteria [73]. These patients have less butyrate-producing bacteria like *Blautia*, *Coprococcus* and *Roseburia* when compared to healthy individuals [74]. A recent study has linked a worsening of disease and elevated amounts of *Eubacterium eligens*, *Eubacterium rectale* and *Eubacterium hallii* [75]. These patients have lower amounts of *Prevotella* species that are connected with production of GABA and larger amounts of *Enterobacteriaceae* family [72]. Also, antibiotics that alter the microbial diversity can impact inflammation, triggering Parkinson’s disease. It highlights possible neuroimmune-specific mechanisms of gut–brain communication [69].

Notably, Parkinson’s disease occurs more often in patients with inflammatory bowel disease (Crohn’s disease and ulcerative colitis). Microbiota associated with inflammatory bowel disease may have considerable impacts on CNS inflammation [76]. However, it is unclear which mechanism is predominant in this case. A recent study showed that patients with inflammatory bowel disease have a 22% higher risk to develop Parkinson’s disease [76]. Another study demonstrated that patients with Parkinson’s disease have *Helicobacter pylori* more frequently than healthy people (14% vs. 4%). Their data suggest that an eradication of the *Helicobacter pylori* infection significantly improve disease symptoms, independently of the use of medications specific to Parkinson’s disease [77]. Nevertheless, it should be taken into an account that broad-spectrum antibiotics were used during the *Helicobacter pylori* eradication process. Such a treatment kills not only *Helicobacter pylori*, but can also significantly changes gut microbiota patterns.

Knowing gut–brain links, it is proposed, that gut microbial changes can primary initiate Parkinson’s disease pathological changes, triggering neuroinflammatory mechanisms [69].

### 3.10. Alzheimers’s Disease

Data available in the literature suggest that an excessive high-fat diet can alter microbial diversity and increase the risk of developing Alzheimer’s disease [78].

Alzheimer’s disease patients also have pathological changes in their blood–brain barrier, which can activate microglia and lead to a chronic neuroinflammatory state. Increased permeability of intestinal and blood–brain barrier, in turn, may increase deleterious signaling through the gut–brain axis [69].

Several bacterial species, for example, *Bacillus subtilis*, *Escherichia coli*, *Mycobacterium tuberculosis*, *Salmonella enterica*, *Salmonella typhimurium*, and *Staphylococcus aureus*, produce amyloid fibers. It was suggested that these fibers are capable of triggering the peripheral immune system, and, thus, enhance the neuroinflammatory responses in the brain [79]. It was also argued that peripherally-produced microbial amyloid proteins enhance Alzheimer’s disease pathogenesis during their accumulation in the CNS.

There is a notion that *Lactobacillus* spp. and *Bacillus* spp. may secrete neurotransmitter acetylcholine, which is reduced in the CNS of patients with Alzheimer’s disease [80]. This study showed that gut microbiota has a mechanism for affecting cellular and molecular features of Alzheimer’s disease. It has also been shown that intestinal microbiota indirectly influences brain pathologies via different neuroinflammatory mechanisms.

### 3.11. Autism Spectrum Disorders

Autism spectrum disorder is a condition with a characteristic deficit in language and social interactions as well as problems with interpreting social cues. Many recent studies have reported changes of microbiota in patients with autism spectrum disorders.

Gut microbiota in patients with autism is often different with increased levels of *Bacteroides phylum*, *Sutterella* species and toxin-producing *Clostridium histolyticum*, but with reduced amounts of *Firmicutes*, *Veilonellaseae*, *Prevotella* and *Coprococcus* species bacteria. There are speculations that some *Clostridium* species bacteria metabolites induce autism spectrum-like behavior.

Finegold’s research group reported increased counts of *Clostridium perfringens* in autism patients, but they also analyzed the main *Clostridium perfringens* toxins genes, finding that the most common toxin in those patients was the β-2 toxin [82]. Another research group presented statistics about a decreased number of *Clostridium* spp. in combination with lower *Lactobacillus* spp. and an aggressive form of *Candida* spp. This aggressive form is characterized by pseudo-hyphae that facilitate adhesion to the intestinal mucosa. The authors also highlighted a linear correlation between severity of autism symptoms and *Clostridium* spp. presence [83].

Other microbial metabolites in high concentrations may contribute to autism spectrum disorders [69,81].

### 3.12. Probiotics and Microbiota Transfer

Taking into account that gut microbiota impacts many host organs and systems, it would be logical to try to modify it, by using different compounds of proven strains in order to normalize host microflora and, directly or indirectly, influence the clinical course of different diseases.

Probiotics are single or multiple strains containing medicines, which are thought to be beneficial for treating different conditions and even for maintaining a healthy gut environment. However, we still lack data about the best strain or combination of strains for many pathologic conditions. Most commonly, the following strains are used: *Lactobacillus* (i.e., *L. rhamnosus*, *L. acidophilus*, *L. reuteri*, *L. bulgaricus*), *Bifidobacteria* (i.e., *B*. *lactis*, *B. longum*), *Saccharomyces boulardii*, *Streptococcus thermophilus*. There is a great variety of factors, which determine the efficacy of probiotical strain and quality standards, like colony-forming units (which differs widely between the strains), resistance to host environment, product storage etc.

The use of probiotics is assessed in the evidence-based medicine in the following most common clinical scenarios: acute and antibiotic-associated diarrhea, irritable bowel syndrome, *Clostridium difficile*-associated diarrhea and *Helicobacter pylori* eradication. In addition, probiotics could be used in case of ulcerative colitis, NAFLD, hepatic encephalopathy, etc. However, many clinical trials point out that the beneficial effect of probiotical products is heterogenous and often of a modest quality, resulting in low-grade recommendations [84,85,86,87,88,89,90,91,92].

One of the potential options in microbiota transfer is fecal microbiota transplantation (FMT). The main idea of FMT is to revert pathological changes of intestinal microflora to normal healthy microbial diversity and proportion. There are different options for how to spread faeces inside of the gut: oral, enema, capsules and colonoscopy. Nevertheless, determining the best donor and standardization of the protocol still present a challenge. To this date, only one indication shows the best evidence in terms of efficacy using FMT, which is recurrent *Clostridium difficile* infection. In the future, the promising directions for applying FMT could be inflammatory bowel disease, hepatic pathology and neurology [93,94].

## 4. Conclusions

We conclude that microbiota can modulate a variety of mechanisms through the gut–liver axis and gut–brain axis. In addition, there is significant evidence that intestinal microbiota, especially altered, can modulate the pathologies of many diseases (i.e., chronic liver and neurological diseases) through different inflammatory mechanisms. However, many pathophysiological reactions in case of microbial disbyosis are still unclear.

On the other side, a healthy intestinal flora impacts the host health in many positive ways. Nonetheless, at the moment, we are limited in our options to use probiotics or FMT as microbiota modificatives in different clinical scenarios, because scientific data are still limited, and many trials are of modest quality.

Future studies should focus on strategies of how to modulate intestinal microbiota populations in individuals at risk for developing different disease.

## Figures and Tables

**Table 1:** Dysbiotic features in liver and CNS diseases.

**Affected Organ-Liver**	
Dysbiotic features and other changes in microbiota	
Alcoholic liver disease	Decreased -butyrate-producing *Clostridiales* spp. -*Bacteroides* and *Lactobacillus* -*Lachnospiracea* and *Ruminococceae* Increased -pro-inflammatory *Enterobacteriaceae* -*Fusobacteria*
NAFLD/NASH	Decreased -*Prevotella* Increased -*Firmicutes/Nacteroides* ratio -*Bacteroides* and *Ruminococcus* -*Escherichia coli*, *Bacteroides vulgatus* (in stage 3 and 4 fibrosis)
Cirrhosis	Decreased -*Bacteroidetes* and *Firmicutes* -*Lachnospiraceae*, *Ruminococceae* Increased -*Enterobacteriaceae* -*Streptococcus* spp., *Veilonella* species -*Veilonella*, *Megasphera*, *Dialister*, *Atobium*, *Prevotella*
Chronic hepatitis B HBV-related cirrhosis	Decreased ratio of *Bifidobacteriaceae/Enterobacteriaceae*: -low levels of *Bifidobacteria* and *Lactobacillus* -high levels of *Enterococcus* and *Enterobacteriaceae* Decreased *Bacteroidetes* Increased *Proteobacteria*
Chronic hepatitis C	Decreased *Bifidobacterium* Increased *Prevotella* and *Faecalibacterium*
HCC	Decreased -*Lactobacillus* spp. *Bifidobacterium* spp., *Enterococcus* spp. Increased -*Escherichia coli* -*Clostridium*
Hepatic encephalopathy	Production of ammonia and endotoxins by urease-producing bacteria, such as *Klebsiella* and *Proteus*
**Affected organ-CNS**	
ALS	Decreased -*Escherichia coli* -*Firmicutes peptostreptococcus* -*Butyrivibrio fibrisolvens*
Parkinson's disease	Decreased -butyrate producing bacteria such as *Blautia*, *Coprococcus*, *Roseburia* -*Prevotella* Increased -*Eubacterium eligens*, *Eubacterium rectale*, *Eubacterium halii* -*Enterobacteriaceae* -more frequently *Helicobacter pylori*
Alzheimer's disease	Decreased -*Lactobacillus* spp., *Bacillus* spp. Possibly increased -*Bacillus subtilis*, *Escherichia coli*, *Mycobacterium tuberculosis*, *Salmonella enterica*, *Salmonella typhimurium*, and *Staphylococcus aureus*
Autism spectrum disorders	Decreased -*Firmicutes*, *Veilonellaseae*, *Prevotella* and *Coprococcus* species Increased -*Bacteroides*, *Sutterella* spp., toxin-producing *Clostridium histolyticum*
